# Prediction of clinical manifestations of transurethral resection syndrome by preoperative ultrasonographic estimation of prostate weight

**DOI:** 10.1186/1471-2490-14-67

**Published:** 2014-08-16

**Authors:** Atsushi Fujiwara, Junko Nakahira, Toshiyuki Sawai, Teruo Inamoto, Toshiaki Minami

**Affiliations:** 1 Department of Anesthesiology, Osaka Medical College, 2-7 Daigaku-machi, Takatsuki, Osaka 569-8686, Japan; 2 Department of Urology, Osaka Medical College, 2-7 Daigaku-machi, Takatsuki, Osaka 569-8686, Japan

**Keywords:** TUR syndrome, Hyponatremia, Transurethral resection of prostate

## Abstract

**Background:**

This study aimed to investigate the relationship between preoperative estimated prostate weight on ultrasonography and clinical manifestations of transurethral resection (TUR) syndrome.

**Methods:**

The records of patients who underwent TUR of the prostate under regional anesthesia over a 6-year period were retrospectively reviewed. TUR syndrome is usually defined as a serum sodium level of < 125 mmol/l combined with clinical cardiovascular or neurological manifestations. This study focused on the clinical manifestations only, and recorded specific central nervous system and cardiovascular abnormalities according to the checklist proposed by Hahn. Patients with and without clinical manifestations of TUR syndrome were compared to determine the factors associated with TUR syndrome. Receiver operating characteristic curve analysis was used to determine the optimal cutoff value of estimated prostate weight for the prediction of clinical manifestations of TUR syndrome.

**Results:**

This study included 167 patients, of which 42 developed clinical manifestations of TUR syndrome. There were significant differences in preoperative estimated prostate weight, operation time, resected prostate weight, intravenous fluid infusion volume, blood transfusion volume, and drainage of the suprapubic irrigation fluid between patients with and without clinical manifestations of TUR syndrome. The preoperative estimated prostate weight was correlated with the resected prostate weight (Spearman’s correlation coefficient, 0.749). Receiver operator characteristic curve analysis showed that the optimal cutoff value of estimated prostate weight for the prediction of clinical manifestations of TUR syndrome was 75 g (sensitivity, 0.70; specificity, 0.69; area under the curve, 0.73).

**Conclusions:**

Preoperative estimation of prostate weight by ultrasonography can predict the development of clinical manifestations of TUR syndrome. Particular care should be taken when the estimated prostate weight is > 75 g.

## Background

Benign prostatic hyperplasia (BPH) is common in elderly men. The risk of BPH increases with age, approaching 50% by the age of 60 years and 90% by the age of 85 years [1]. Numerous therapeutic options are available for BPH, including pharmacological treatment, minimally invasive surgery, and open prostatectomy. Preoperative ultrasonography is often performed to confirm the diagnosis of BPH and to measure the shape, volume, and structure of the prostate.

Transurethral resection of the prostate (TURP) is a standard surgical treatment for BPH. Non-conductive irrigation fluid is used during TURP to maintain good visibility of the operating field during resection of the prostate with monopolar cutting diathermy. The non-conductive irrigation fluid contains no electrolytes, and absorption of this hypotonic solution into the bloodstream can cause fluid overload and dilutional hyponatremia, resulting in adverse cardiovascular and central nervous system effects. Transurethral resection (TUR) syndrome is usually defined as a serum sodium level of < 125 mmol/l combined with clinical cardiovascular or neurological manifestations [2,3]. However, the clinical manifestations can also occur with a serum sodium level of > 125 mmol/l. Because of the multifactorial pathophysiology of TUR syndrome, few studies have used a clear and consistent definition of this condition. This study used the severity score for TUR syndrome proposed by Hahn, which is based on a checklist of central nervous system and cardiovascular abnormalities (Table 1) [4].

**Table 1:** Severity score checklist

	**Severity score**		
	**1**	**2**	**3**
Circulatory			
Chest pain	Duration < 5 min	Duration > 5 min	Repeated attacks
Bradycardia	HR decrease 10–20 bpm	HR decrease > 20 bpm	Repeated decreases
Hypertension	SAP up 10–20 mmHg	SAP up > 30 mmHg	Score (2) for 15 min
Hypotension	SAP down 30–50 mmHg	SAP down > 50 mmHg	Repeated drops > 50 mmHg
Poor urine output	Diuretics needed	Repeated use	Diuretics ineffective
Neurological			
Blurred vision	Duration < 10 min	Duration > 10 min	Transient blindness
Nausea	Duration < 5 min	Duration 5–120 min	Intense or > 120 min
Vomiting	Single instance	Repeatedly, < 60 min	Repeatedly, > 60 min
Uneasiness	Slight	Moderate	Intense
Confusion	Duration < 5 min	Duration 5–60 min	Duration > 60 min
Tiredness	Patient says so	Objectively exhausted	Exhausted for > 120 min
Consciousness	Mildly depressed	Somnolent < 60 min	Needs ventilator
Headache	Mild	Severe < 60 min	Severe > 60 min

A checklist used to define and score the clinical manifestations of TUR syndrome [2].

HR, heart rate; SAP, systolic arterial pressure.

The theoretical risk factors for TUR syndrome include patent prostatic sinuses, high irrigation pressure, prolonged operation time, and use of hypotonic irrigation fluid [5]. It was reported that 77% of patients undergoing TURP had significant pre-existing medical conditions, and that resection time > 90 min, estimated prostate weight > 45 g, acute urinary retention, age > 80 years, and African descent were associated with increased morbidity [2,5]. This study aimed to determine the risk factors for development of the clinical manifestations of TUR syndrome, and to investigate whether these clinical manifestations could be predicted by preoperative estimation of prostate weight by ultrasonography.

## Methods

After obtaining approval from the Ethical Committee of Osaka Medical College (reference number: 898), patients at our institution were informed of this retrospective observational study on a bulletin board. We retrospectively reviewed the records of patients who underwent TURP under combined spinal and epidural anesthesia from April 2006 to March 2011. Spinal anesthesia was administered at L2/3, L3/4, or L4/5, and the epidural space was catheterized at L1/2 or L2/3. Intrathecal bupivacaine hydrochloride (hyperbaric, 0.5%, 2.0–3.0 ml) was administered to achieve a T10 sensory level. Patients who underwent surgery under general anesthesia because spinal anesthesia failed were excluded from the study. If the sensory level was lower than T10, or the duration of surgery was > 90 min, 0.375% ropivacaine hydrochloride (3.0–5.0 ml) was administered via the epidural catheter. Postoperative analgesia was provided by continuous epidural infusion of 0.2% ropivacaine at 2–5 ml/h. All surgical procedures were performed using an electronic resectoscope with a monopolar view, by surgeons with the same qualifications and clinical experience. D-sorbitol 3% was used as the non-conductive irrigation fluid, with the bags positioned 90 cm above the operating table. Hemodynamic monitoring included non-invasive measurement of systolic and diastolic blood pressure every 2 min and continuous monitoring of the heart rate, electrocardiogram, and pulse oximetry. Patients with bleeding disorders, renal insufficiency, and contraindications to spinal anesthesia were excluded. All patients received intravenous infusion of lactated Ringer’s solution before spinal anesthesia.

Clinical manifestations of TUR syndrome were scored using the checklist proposed by Hahn, which recorded central nervous system abnormalities (such as nausea, vomiting, restlessness, and coma) and intra- or postoperative cardiovascular abnormalities (Table 1) [4]. At least one neurological and one cardiovascular abnormality were required for patients to be included in the TUR syndrome group. For cardiovascular abnormalities, such as hypertension (systolic blood pressure > 30% above the baseline), hypotension (systolic blood pressure < 80 mmHg), bradycardia, and arrhythmia, immediate treatment was administered to avoid further deterioration. For systolic blood pressure < 80 mmHg, 4 mg ephedrine hydrochloride was administered intravenously. The medical and nursing staff closely monitored patients during and after the procedure to detect and treat complications, and to evaluate the severity of clinical manifestations of TUR syndrome. All anesthetic charts included detailed records of patient status. Manifestations of TUR syndrome were differentiated from manifestations of a vasovagal reflex caused by filling of the bladder or by the epidural and spinal anesthesia.

**Figure 1:**
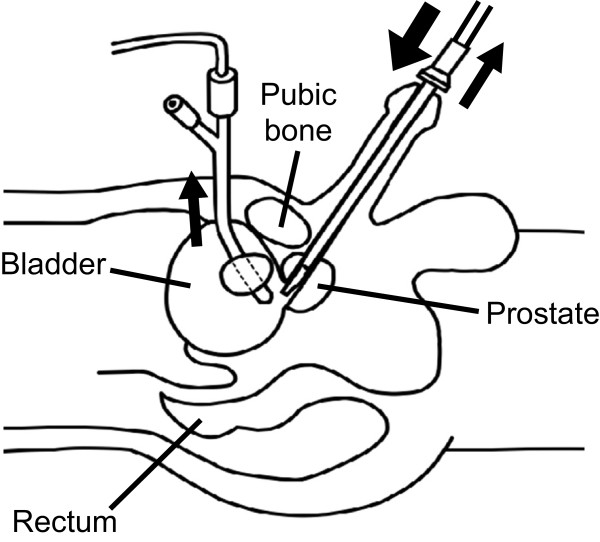
**Continuous drainage of irrigation fluid.** Irrigation fluid drains from the bladder via the resectoscope and the drainage catheter inserted through a suprapubic cystostomy. This image was reproduced from reference [6].

Patients were divided into groups with and without clinical manifestations of TUR syndrome, and potential risk factors were compared between the two groups. Patient characteristics, dose of regional anesthetic, duration of surgery, resected prostate weight, intravenous infusion volume, blood transfusion volume, and whether the irrigation fluid was continuously drained through a suprapubic pigtail drainage catheter (C. R. Bard, Karlsruhe, Germany; Figure 1) [6] were recorded. Potential manifestations of TUR syndrome were treated to prevent further complications. Blood sampling was performed at the discretion of the anesthesiologist and surgeon. The anesthesiologist determined whether clinical abnormalities were caused by TUR syndrome or by the anesthesia or sedation.

The prostate size was estimated preoperatively by transrectal longitudinal ultrasonography with a real-time linear scanner and 5.0-MHz transducer (Hitachi Aloka Medical Ltd, Tokyo, Japan). The maximal length (A) and maximal width (B) of the prostate were measured. Assuming that the prostate is ellipsoid in shape in patients with BPH, the volume (V) was calculated according to the formula V = πAB^2^ / 6. The prostate weight was assumed to be approximately equal to V as the specific gravity of prostatic tissue in patients with BPH is 1.05–1.06 g/cm^3^[7]. The hand-rolling method was used to measure the resected prostate weight after TURP [8].

The Mann–Whitney U test and unpaired t-test were used to compare potential risk factors for TUR syndrome, including age, prostate weight, and operating time [9], between patients with and without clinical manifestations of TUR syndrome. Spearman’s rank correlation coefficient was used to evaluate the relationship between the preoperative estimated prostate weight and the resected prostate weight. Receiver operator characteristic curve analysis was performed to determine the predictive value and optimal cutoff point of preoperative estimated prostate weight for prediction of the development of clinical manifestations of TUR syndrome. A *p* value of < 0.05 was considered statistically significant. All analyses were performed using GraphPad Prism version 5.0 for Mac (GraphPad Software, San Diego, CA, USA).

## Results

**Figure 2:**
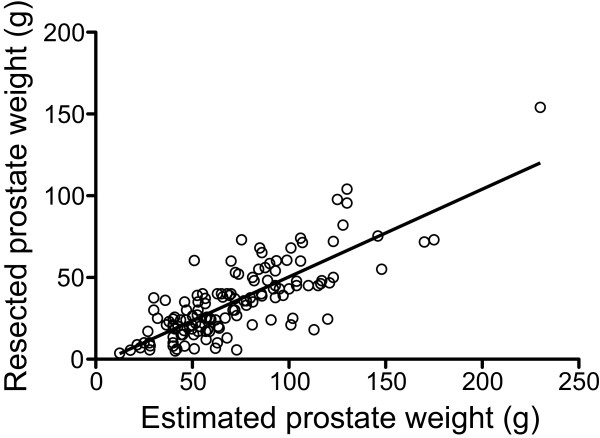
Correlation between estimated prostate weight and resected prostate weight.

**Figure 3:**
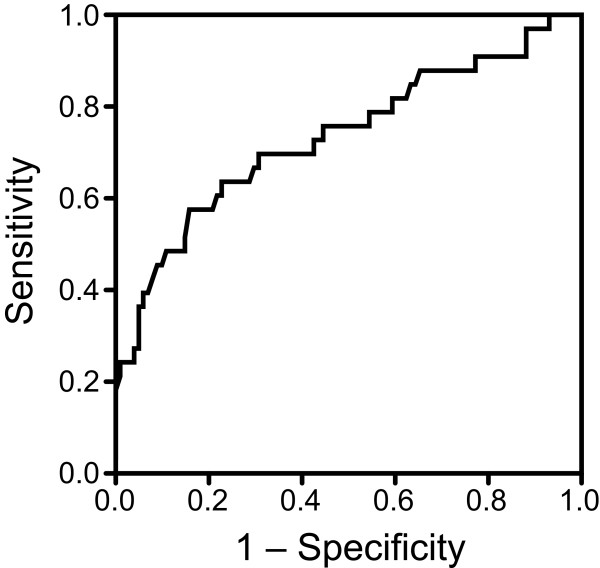
Receiver operating characteristic curve showing the ability of estimated prostate weight to predict TUR syndrome.

A total of 167 patients were included in this study, of which 42 developed clinical manifestations of TUR syndrome (24.2%; 95% confidence interval, 18.5%–31.8%). The majority of initial cardiovascular abnormalities were either hypertension with reflex bradycardia, or sudden hypotension. There were no significant differences in preoperative characteristics between patients with and without clinical manifestations of TUR syndrome, except for the preoperative estimated prostate weight (Table 2). There were significant differences in the duration of surgery, resected prostate weight, intravenous infusion volume, blood transfusion volume, and continuous drainage of irrigation fluid via suprapubic cystostomy between patients with and without clinical manifestations of TUR syndrome (Table 3). The postoperative serum sodium level and hemoglobin concentration were significantly lower in patients with than without clinical manifestations of TUR syndrome. All patients who developed clinical manifestations of TUR syndrome had a severity score of ≥ 2 according to Hahn’s checklist at the end of surgery (Table 1). Patients with a score of ≥ 3 required additional intravenous anesthetic agents such as propofol or midazolam. One patient developed severe hyponatremia and required tracheal intubation to manage his cardiovascular and neurological abnormalities. One patient without clinical manifestations of TUR syndrome developed postoperative esophageal hemorrhage from ruptured esophageal varices, which was judged to be unrelated to the TURP procedure. One patient without intraoperative manifestations of TUR syndrome developed postoperative nausea and vomiting. All blood transfusions were autologous, except in one of the patients who developed clinical manifestations of TUR syndrome who received an allogeneic transfusion.The preoperative estimated prostate weight was correlated with the resected prostate weight (Spearman’s correlation coefficient, 0.749; Figure 2). Receiver operator characteristic curve analysis showed that the optimal cutoff value of estimated prostate weight for the prediction of clinical manifestations of TUR syndrome was 75 g (sensitivity, 0.70; specificity, 0.69; area under the curve, 0.73; Figure 3).

**Table 2:** Patient characteristics

	**Symptomatic (n = 42)**	**Asymptomatic (n = 125)**	***p *** **value**
Age, years	72 ± 8	70 ± 7	0.202
Height, cm	164.6 ± 6.1	164.6 ± 5.8	0.989
Body weight, kg	63.2 ± 10.6	63.0 ± 9.2	0.925
Diabetes mellitus	2 (4.8%)	9 (7.2%)	0.732
Hypertension	3 (7.1)	9 (7.2%)	1.000
Arrhythmia	0 (0.0%)	1 (0.8%)	1.000
Preoperative blood data			
Creatinine, mg/dl	0.9 ± 0.2	0.9 ± 0.2	0.718
BUN, mg/dl	15.7 ± 5.9	15.2 ± 4.0	0.912
Sodium, mmol/l	140.5 ± 2.2	140.5 ± 2.4	0.942
Hemoglobin, g/dl	13.6 ± 1.6	14.1 ± 1.5	0.321
Hematocrit,%	39.5 ± 4.5	40.7 ± 4.9	0.218
Estimated prostate weight, g	99.0 ± 45.6	64.6 ± 26.4	< 0.001

Data are expressed as mean ± SD or number (%). Symptomatic = the presence of central nervous system abnormalities such as nausea, vomiting, restlessness, pain, confusion, and coma together with intra- or postoperative cardiovascular abnormalities; BUN = blood urea nitrogen.

**Table 3:** Intraoperative and postoperative data

**Parameter**	**Symptomatic (n = 42)**	**Asymptomatic (n = 125)**	***p *** **value**
Continuous drainage of irrigation fluid	23 (54.8%)	17 (13.6%)	< 0.001
Intrathecal 0.5% bupivacaine, ml	2.4 ± 0.4	2.5 ± 0.5	0.869
Resected prostate weight, g	52.3 ± 29.7	29.8 ± 18.7	< 0.001
Operation time, min	101 ± 34	71 ± 26	< 0.001
Operation time > 90 min	25 (59.5%)	32 (25.6%)	< 0.001
Total infusion volume, ml	903 ± 598	578 ± 284	0.002
Symptoms	42 (100.0%)	-	NA
Restlessness	24 (57.1%)	-	NA
Vomiting	14 (33.3%)	-	NA
Nausea	22 (52.3%)	-	NA
Pain	17 (40.5%)	-	NA
Confusion	11 (26.2%)	-	NA
Blood transfusion	26 (61.9%)	24 (19.2%)	< 0.001
Diuretics	6 (14.3%)	1 (0.8%)	0.003
Saline infusion	0 (0.0%)	1 (0.8%)	0.001
Postoperative blood data			
Creatinine, mg/dl	0.9 ± 0.3	0.9 ± 0.2	0.943
BUN, mg/dl	12.4 ± 5.0	12.8 ± 4.1	0.441
Sodium, mmol/l	133.4 ± 7.9	138.0 ± 3.8	< 0.001
Hemoglobin, g/dl	11.0 ± 1.8	12.8 ± 1.6	< 0.001
Hematocrit,%	32.0 ± 5.6	37.4 ± 4.6	< 0.001

Data are expressed as mean ± SD or number (%). Symptomatic = the presence of central nervous system abnormalities such as nausea, vomiting, restlessness, pain, confusion, and coma together with circulatory intra- or postoperative cardiovascular abnormalities; BUN = blood urea nitrogen.

## Discussion

The incidence of TUR syndrome was higher in our cohort than in previous studies, which reported rates from 0.5% to 10.5% [2,3,7]. This can be explained by the varying definitions of TUR syndrome used. Many previous studies defined TUR syndrome as a serum sodium level of ≤125 mmol/l after TURP with two additional abnormalities such as nausea, vomiting, bradycardia, hypotension, chest pain, mental confusion, anxiety, paresthesia, or visual impairment [3]. The proportion of patients in our study that met this definition based on the sodium level was 13.2%. This study focused on the clinical manifestations of TUR syndrome, regardless of the serum sodium level. The clinical manifestations included central nervous system abnormalities such as nausea, vomiting, restlessness, and coma, and intra- and postoperative cardiovascular abnormalities, according to the checklist proposed by Hahn [4]. Some of our patients with neurological and cardiovascular manifestations of TUR syndrome did not have a serum sodium level of < 125 mmol/l. Patients with a severity score of 1 were treated to avoid further deterioration, regardless of the serum sodium level.

Our results show an optimal cutoff value for estimated prostate weight of 75 g to predict the development of clinical manifestations of TUR syndrome, which is heavier than previously suggested weights. Previous studies reported that the most significant risk factors for TUR syndrome were an operation time of > 90 min, a heavier prostate weight such as > 45 g, acute urinary retention, and age > 80 years [2]. These factors increase the risk of TUR syndrome because of the larger quantity of irrigation fluid absorbed. Technical advances and resection speeds of 0.5–0.9 g/min have not resulted in a significant reduction in the incidence of TUR syndrome [10]. This study was conducted in our specialized college hospital that is associated with satellite hospitals, and the patients who undergo TURP at our hospital tend to have relatively large prostate glands, resulting in longer operation times and a higher incidence of TUR syndrome. Some centers may perform open prostatectomy in patients with large prostate glands, but TURP is the standard procedure for such cases in Japan.

This is the first study to investigate the relationship between prostate weight and the development of clinical manifestations of TUR, regardless of serum sodium levels. Preoperative ultrasonography is commonly used to diagnose BPH and to estimate prostate weight. In this study, there was a strong correlation between the preoperative estimated prostate weight on ultrasonography and the resected prostate weight, indicating that preoperative estimation of prostate weight by ultrasonography may be useful for predicting the risk of TUR syndrome. The optimal cutoff value of estimated prostate weight to predict the development of clinical manifestations of TUR syndrome was 75 g. However, there is also a risk of TUR syndrome when resecting prostates of lower weights. Akata *et al.* reported that changes in the serum sodium level during TURP correlated with incision of the capsular veins and prostatic sinuses, but not with operation time [11]. It is important to carefully monitor patients for the development of TUR syndrome, especially patients with larger prostates, and we recommend measurement of the serum sodium level during and after surgery.

In this study, continuous drainage of irrigation fluid through a suprapubic cystostomy was found to be a risk factor for TUR syndrome. Our previous study also found this to be an important risk factor for TUR syndrome in older patients [12]. Such continuous drainage of irrigation fluid facilitates the removal of debris, blood, and clots from the operating field. Blood clots and debris may obstruct the drainage catheter, thereby raising the fluid pressure and increasing the volume absorbed [13]. Drainage catheters with small diameters may be less effective than catheters with larger diameters. A number of patients in this study were noted to have abdominal swelling caused by leakage of irrigation fluid through the drainage site into the extraperitoneal space and abdominal cavity. When this occurs, extracellular electrolytes diffuse into the accumulated irrigation fluid [14], resulting in dilutional hyponatremia and increasing the risk of TUR syndrome. The hyponatremia is most pronounced at 2–4 h after surgery, but may go undetected until the next day [15].

Patients with a preoperative prostate weight of > 75 g should receive additional treatment to reduce the risk of TUR syndrome, such as blood transfusion, intravenous diuretics, and saline infusion. It is generally recommended that surgery should be performed under regional anesthesia when there is an increased risk of TUR syndrome, as this enables early detection of gross changes in mental status, but this is not universally accepted [16]. TUR syndrome can have many causes and the clinical manifestations may be vague, making early detection difficult. It is therefore important to identify the risk factors for TUR syndrome to increase vigilance among medical and nursing staff and enable early intervention.

This study is limited by its retrospective, observational design. However, the patient details and timing of blood tests were carefully evaluated using data recorded in the comprehensive preoperative and anesthetic records to ensure accuracy. A further prospective study with a larger study population should be conducted to verify our findings.

## Conclusions

In this study, preoperative estimation of prostate weight by ultrasonography could predict the development of clinical manifestations of TUR syndrome. When the preoperative estimated prostate weight is > 75 g, patients should be monitored closely and appropriate intervention should be planned.

## Competing interests

The authors declare that they have no competing interests.

## Authors’ contributions

AF participated in the design and coordination of the study and helped to draft the manuscript. JN made substantial contributions to the conception and design of the study and the acquisition of data, and drafted the manuscript and tables. TS performed the statistical analyses and revised the manuscript critically for important intellectual content. TI made substantial contributions to the conception of the study and helped to correct the manuscript. TM made substantial contributions to the conception of the study and helped to draft the manuscript. All authors read and approved the final manuscript.

## Pre-publication history

The pre-publication history for this paper can be accessed here:

http://www.biomedcentral.com/1471-2490/14/67/prepub
